# Exercise-induced albuminuria increases over time in individuals with impaired glucose metabolism

**DOI:** 10.1186/s12933-020-01058-9

**Published:** 2020-06-15

**Authors:** Rafael Y. Brzezinski, Limor Friedensohn, Itzhak Shapira, David Zeltser, Ori Rogowski, Shlomo Berliner, Ayelet Grupper, Shani Shenhar-Tsarfaty

**Affiliations:** 1 Department of Internal Medicine “C”, “D” and “E”, Tel Aviv Sourasky Medical Center, 6 Weizmann Street, 64239 Tel Aviv, Israel; 2 Sackler Faculty of Medicine, Tel Aviv University, Tel Aviv, Israel; 3 Neufled Cardiac Research Institute, Sackler Faculty of Medicine, Tel Aviv University, Tel Aviv, Israel; 4 Tamman Cardiovascular Research Institute, Sheba Medical Center, Tel Hashomer, Israel; 5 Nephrology Department, Tel-Aviv Sourasky Medical Center, Tel Aviv, Israel

**Keywords:** Albuminuria, Blood glucose, Diabetes, Endothelial dysfunction, Exercise

## Abstract

**Background:**

Exercise induced albuminuria (EiA) is elevated in patients with metabolic dysfunction and diabetes, and may serve as an early biomarker for endothelial dysfunction and “kidney reserve”. However, the change in EiA levels over time and its interaction with metabolic dysfunction and glucose metabolism has never been studied. Therefore, we sought to determine EiA levels over time in a cohort of individuals attending a routine annual health survey.

**Methods:**

We prospectively enrolled 412 patients attending an annual healthy survey at our Medical Center. We collected urine samples for albumin and creatinine measurements before and immediately after completing an exercise stress test, along with multiple physiologic and metabolic parameters. Participants returned to a second follow up visit after a mean follow up period of 3 years (± 1.7 SD).

**Results:**

Patients with diagnosed diabetes and subjects with HbA1c ≥ 6.5% significantly increased their EiA over time (median [IQR] change between visits = 19.5 [− 10.4–56.1] vs. − 1.1 [− 12.7–4.9] (p = 0.049) for diabetics vs non-diabetics respectively). Moreover, a diabetes diagnosis was significantly associated with a high increase in EiA over time (top 10th percentile) even after adjusting for age, BMI, eGFR, METs, self-reported history of heart disease, systolic and diastolic blood pressure; OR = 4.4 (1.01–19.3 95% CI) (p = 0.049). Finally, elevated fasting blood glucose (≥ 100 mg/dl) was the strongest and only significant predictor for a greater increase in EiA over time after adjusting for all five metabolic syndrome components; blood glucose, waist circumference, blood triglycerides, HDL cholesterol, and BP criteria; OR = 4.0 (1.6–9.8 95% CI) (p < 0.01).

**Conclusions:**

Patients with diabetes and/or elevated fasting blood glucose increase their exercise-induced urinary albumin excretion over time. The ability of EiA to predict major clinical outcomes in patients with and without diabetes needs to be determined in future studies.

## Background

Diabetes and impaired glucose metabolism lead to endothelial dysfunction and kidney injury [1]. Excessive urinary albumin excretion reflects damage to the kidney’s vasculature bed and precedes kidney failure [2]. Moreover, even moderately increased levels of urinary albumin excretion (measured by a spot urinary albumin to creatine ratio test (UACR)) correlate with elevated risk for cardiovascular disease (CVD) [3–5].

Exercise induced albuminuria (EiA) appears earlier and may be a more sensitive biomarker for kidney endothelial damage [6]. We and others have shown that elevated EiA levels are associated with metabolic syndrome [7], hypertension [8], and diabetes [9, 10]. However, the change in an individual’s EiA levels over time and its interaction with metabolic dysfunction has never been studied.

A recent report showed that changes in rest UACR over time modifies the risk of major clinical outcomes and mortality in diabetic patients [11]. Accordingly, we hypothesize that measuring changes in EiA over time should enable earlier risk stratification.

Here, we sought to determine the change in EiA levels over time in a cohort of diabetic and apparently healthy individuals, and to reveal the factors responsible for “abnormal” changes.

## Methods

### Study cohort

We prospectively enrolled consecutive patients attending a routine annual health survey at the Tel-Aviv Medical Center Inflammation Survey (TAMCIS), a registered databank of the Israeli Ministry of Justice [12–16]. Subjects underwent a physician’s interview and examination, blood and urine tests, and an exercise stress test. The study was approved by the local Ethics committee and informed consent was obtained from all participants.

We collected urine samples for albumin and creatinine measurements from participants before and after an exercise stress test and analyzed data collected from patients attending at least two consecutive visits at TAMCIS. Out of 433 subjects with at least two consecutive visits, we excluded 19 subjects due to lack of urinary tests (before or after the exercise test, on one or more visits). Patients with diagnosed kidney failure (eGFR < 30 ml/min/1.73 m^2^) and patients with a prior incidence of stroke were also excluded. The final study cohort comprised 412 participants. Population characteristics are presented in Table 1.

**Table 1:** Population characteristics (Total N = 412)

Characteristic	Normal EiA on both visits	Elevated EiA only on first visit	Elevated EiA only on second visit	Elevated EiA on both visits	p
N	269	58	37	48	
Age, years	48.7 (9.4)	47.3 (10.0)	49.3 (8.4)	47.0 (8.3)	0.48
Men (%)	43 (16)	8 (14)	6 (16)	5 (10)	0.78
BMI, kg/m^2^	25.7 (3.8)	27.0 (4.6)	26.8 (3.2)	29.5 (5.1)	< 0.01
Δ BMI, kg/m^2^	− 0.4 (3.6)	− 0.9 (4.3)	− 0.1 (3.4)	− 1.0 (2.9)	0.55
Systolic BP, mmHg	125.7 (13.5)	127.5 (13.1)	125.0 (14.0)	128.1 (14.9)	0.55
Δ Systolic BP, mmHg	0.5 (13.4)	0.2 (11.1)	− 1.1 (11.3)	3.5 (14.1)	0.39
Diastolic BP, mmHg	78.7 (8.8)	80.9 (9.1)	78.5 (9.7)	82.2 (9.2)	0.04
Δ Diastolic BP, mmHg	− 0.5 (10.6)	− 0.4 (13.5)	− 0.9 (8.5)	− 2.2 (10.6)	0.78
Rest heart rate, BPM	68.4 (11.1)	68.6 (12.7)	66.5 (12.7)	68.6 (13.3)	0.81
Δ Rest heart rate, BPM	0.5 (11.6)	1.1 (13.4)	− 4.5 (10.8)	1.2 (12.4)	0.10
METs	12.0 (2.2)	12.2 (2.2)	11.8 (2.4)	12.9 (2.2)	0.12
Δ METs	3.2 (52.6)	− 0.3 (1.9)	0.5 (2.0)	− 0.1 (1.6)	0.94
Fasting blood glucose, mg/dl	86.9 (8.4)	89.4 (15.5)	88.9 (19.2)	92.0 (19.5)	0.04
Δ Fasting blood glucose, mg/dl	− 0.6 (9.3)	− 3.4 (10.1)	1.0 (21.8)	− 1.0 (12.6)	0.26
HbA1c, %	5.4 [5.2, 5.7]	5.5 [5.3, 5.8]	5.5 [5.3, 5.7]	5.6 [5.2, 5.8]	0.35
Δ HbA1c, %	0.1 (0.4)	0.1 (0.3)	0.1 (0.3)	0.1 (0.3)	0.85
Total cholesterol, mg/dl	185.2 (31.6)	187.6 (32.1)	181.3 (38.2)	186.9 (30.7)	0.81
Δ Total cholesterol, mg/dl	− 1.1 (27.2)	− 5.2 (22.4)	− 10.1 (21.4)	− 4.5 (28.7)	0.20
High-density lipoprotein-cholesterol, mg/dl	53.1 (13.1)	52.1 (16.3)	52.6 (18.2)	51.8 (16.9)	0.92
Δ High-density lipoprotein-cholesterol, mg/dl	− 0.8 (6.9)	− 0.7 (10.9)	0.1 (7.2)	− 1.0 (9.1)	0.92
Low-density lipoprotein-cholesterol, mg/dl	111.6 (25.9)	111.0 (26.4)	107.8 (30.5)	109.1 (24.7)	0.82
Δ Low-density lipoprotein-cholesterol, mg/dl	− 0.7 (24.0)	− 4.0 (18.6)	− 8.9 (16.1)	− 3.7 (23.4)	0.18
Triglycerides, mg/dl	109.2 (83.0)	122.6 (64.6)	104.5 (47.6)	130.1 (62.4)	0.21
Δ Triglycerides, mg/dl	− 3.8 (61.4)	− 2.8 (52.4)	− 6.5 (39.6)	1.6 (44.1)	0.92
eGFR, ml/min/1.73 m^2^	77.4 (12.3)	79.2 (14.0)	79.1 (11.1)	80.0 (11.7)	0.44
Δ eGFR, ml/min/1.73 m^2^	5.9 (10.4)	4.7 (11.9)	4.9 (7.5)	2.9 (9.8)	0.28
Rest UACR, mg/g	3.2 [1.1, 6.3]	5.8 [1.7, 13.5]	3.7 [1.6, 8.5]	5.7 [2.6, 16.4]	< 0.01
Δ Rest UACR, mg/g	− 0.2 [− 3.2, 1.5]	− 1.8 [− 7.8, 1.1]	− 0.4 [− 4.2, 1.9]	− 0.3 [− 5.4, 4.4]	0.19
EiA, mg/g	1.1 [− 1.2, 5.1]	33.9 [25.9, 66.6]	2.8 [− 3.5, 8.2]	71.8 [38.3, 154.6]	< 0.01
Δ EiA, mg/g	− 0.1 [− 4.2, 3.0]	− 31.2 [− 62.1, − 21.1]	41.1 [29.0, 80.9]	− 17.5 [− 87.6, 30.0]	< 0.01
Blood creatinine, mg/dl	1.1 (0.2)	1.1 (0.6)	1.1 (0.1)	1.1 (0.1)	0.46
Δ Blood creatinine, mg/dl	− 0.1 (0.1)	− 0.1 (0.6)	− 0.1 (0.1)	− 0.1 (0.1)	0.31
Blood Albumin, g/l	45.2 (2.3)	45.5 (2.8)	45.5 (1.8)	45.8 (2.6)	0.32
Δ Blood Albumin, g/l	− 0.9 (2.3)	− 1.0 (2.5)	− 1.3 (2.2)	− 1.0 (2.3)	0.78
Diabetes diagnosis (%)	3 (1)	3 (5)	4 (11)	3 (6)	0.01
Pre-diabetes diagnosis (%)	39 (15)	15 (26)	6 (16)	12 (26)	< 0.01
History of heart disease	12 (5)	4 (7)	4 (11)	5 (10)	0.25
Antihypertensive medications (%)	28 (10)	14 (24)	6 (16)	9 (19)	0.03
ACE inhibitors/ARBs (%)	21 (8)	10 (17)	5 (14)	7 (15)	0.11
Lipid lowering medications (%)	33 (12)	11 (19)	10 (27)	12 (25)	0.03

*BMI* body mass index, *HbA1c* hemoglobin A1c, *BPM* beats per minute, *BP* blood pressure*, METs* metabolic equivalents, *UACR* urinary albumin to creatinine ratio, *EiA* exercise induced albuminuria, *eGFR* estimated glomerular filtration rate, *ACE inhibitors* angiotensin converting enzyme inhibitors, *ARBs* angiotensin receptor blockers

Values are presented as mean (SD), or median [interquartile range] for irregular distributed parameters

Elevated EiA: > 20 mg/g (see details in “Methods” section)

Δ refers to the change in a characteristic between visits = measurement on second visit − measurement on first visit

### Study procedures

Blood samples were drawn upon the participants’ arrival to the center after a 12-h overnight fast. An Exercise ECG stress test was preformed according to the Bruce protocol [17]. ECG results were manually reviewed on the spot by a cardiologist. Only after the morning blood test participants were allowed to drink, and after the exercise test they were invited to eat breakfast.

Urine tests were analyzed by an ADVIA chemistry analyzer (Siemens Healthcare Diagnostics, Tarrytown, NY), which is an improved albumin detection method with an extended analytic range of 3–420 mg/l and coefficient of variation of 2% [18]. It is accepted that a test for UACR is the standard method for assessing proteinuria using a spot examination, as advised by National Kidney Foundation Kidney Disease Outcomes Quality Initiative guidelines [19]. Therefore, we performed calculation of the UACR with a standard cutoff of 30 mg/g for moderately increased albuminuria. eGFR values were calculated using the CKD-EPI formula [20].

Separation of HbA1c from non-glycated hemoglobin of whole blood samples in EDTA was done by Tosoh’s G7 HPLC (Tosoh Bioscience, Inc. San Francisco, CA). The concentrations of glycated hemoglobin (HbA1c) and the concentration of total hemoglobin were measured, and the ratio reported as % HbA1c. HbA1c levels were categorized into three states, healthy; < 5.7%, pre-diabetic; 5.7–6.4% and diabetic; ≥ 6.5% according to the American Diabetes Association (ADA) guidelines [21].‬‬‬‬‬‬‬‬‬‬‬‬‬‬‬‬‬‬‬‬‬‬‬‬‬‬‬‬‬‬‬‬‬‬‬‬‬‬‬‬‬‬‬‬‬‬‬

### EiA measurement

We measured urinary albumin and creatinine before and after the exercise stress test. EiA was defined as the change in albumin excretion following exercise (exercise albumin- rest albumin) divided by the rest urinary creatine measurement.

Elevated EiA was defined as > 20 mg/g as this was the lower bound of the top quartile of the cohort. Similar cut-off values have been shown to be associated with metabolic dysfunction and abnormal cardiac findings in our previous reports [8, 22].

### Metabolic syndrome definition

Evaluation and diagnosis of metabolic syndrome (and it’s components) was performed based on the joint interim statement of the International Diabetes Federation Task Force on Epidemiology and Prevention; National Heart, Lung, and Blood Institute; American Heart Association; World Heart Federation; International Atherosclerosis Society; and International Association for the Study of Obesity [23]. Briefly, elevated waist circumference was defined as ≥ 94 cm (37 in.) in men and ≥ 80 (31.5 in.) in women, as recommended for individuals of European and Middle Eastern descent. Elevated triglycerides were defined as ≥ 150 mg/dl (1.7 mmol/l) or on drug treatment for elevated triglycerides. Reduced high-density lipoprotein-cholesterol (HDL) was defined as < 40 mg/dl (1.0 mmol/l) in men and < 50 mg/dl (1.3 mmol/l) in women. Elevated blood pressure was defined as ≥ 130 mmHg for systolic blood pressure or ≥ 85 mmHg for diastolic blood pressure or on antihypertensive drug treatment in a patient with a history of hypertension. Elevated fasting glucose was defined as ≥ 100 mg/dl (5.55 mmol/l). The diagnosis of metabolic syndrome was based on the existence of at least three abnormal findings out of the five mentioned above.

Blood pressure was recorded as the mean of 2 seated measurements. Hypertension was defined as systolic blood pressure of 140 mmHg or higher, diastolic blood pressure of 90 mmHg or higher, or the use of antihypertensive medications. Diagnosed diabetes mellitus was defined as a self-reported physician diagnosis of diabetes or current use of diabetic medications.

### Statistical analysis

All continuous variables are displayed as means (SD) for normally distributed variables or median [interquartile range (IQR)] for variables with abnormal distribution. Categorical variables are displayed as numbers (%) of patients within each group. The different biomarkers in patients with and without diabetes were compared by a Student’s *t*-test for normally distributed variables and by the Mann–Whitney *U*-test for non-normally distributed variables. To assess associations among categorical variables, we used a χ^2^-test. Spearman’s test was used to assess the correlation between EiA levels on the first and second visit.

We used Kruskal–Wallis’s test with Dunn’s test for multiple comparisons to evaluate the difference in EiA according to categorized HbA1c levels.

To identify possible confounders, we performed a multivariate binary logistic regression to predict a high increase in EiA between visits (top 10th percentile; > 30 mg/g). The model was adjusted for the following covariates measured during the participants` first visit: age, diabetes diagnosis, body mass index (BMI), eGFR, self-reported history of heart disease, systolic and diastolic blood pressure at rest and metabolic equivalents (METs). To determine the relative effect of each metabolic syndrome component on the increase in EiA between visits, we used the same model adjusted to all five components (waist criteria, blood pressure criteria, HDL criteria, triglyceride criteria and blood glucose criteria). The same model was then used to predict a high increase in rest UACR between visits (top 10th percentile, > 6 mg/g). We performed an additional binary logistic regression to predict a high increase in EiA adjusted to the following covariates: age, sex and the continuous change between visits in fasting blood glucose, BMI, eGFR, systolic and diastolic blood pressure, and METs.

We used the R statistical package (version 3.3.1, R Foundation for Statistical Computing, Vienna, Austria) along with IBM SPSS Statistics 25.0 statistical package (IBM Corporation, Armonk, New York, USA) and GraphPad Prism version 8.00 (GraphPad Software, La Jolla, CA, USA) for all statistical analysis.

## Results

Baseline characteristics are presented in Table 1. Our study cohort was male dominant (85%) and had relatively low prevalence of pre-existing comorbidities. The mean follow-up period between the subjects' first and second visit was 3 years (± 1.7 SD).

Rest UACR levels were 3.7 mg/g [1.3–8.7] during the first visit and 2.4 mg/g [1.2–5.5] for median [IQR] during the follow up visit. The median change in rest UACR was − 0.4 mg/g [− 4.5 to 1.7] [IQR].

Urinary albumin concentrations were elevated after exercise across all consecutive visits. The median change in urinary albumin from before to after exercise was 2.3 mg/l [− 0.1 to 12.1] [IQR] on the first visit and 1.5 mg/l [0–9.4] [IQR] on the second visit. The exercise induced change in UACR (EiA) was 3.9 mg/g [− 0.2, 21.8] for median [IQR] on the first visit and 2.4 mg/g [0–13.4] during the follow-up visit.

### EiA and metabolic syndrome status

**Fig. 1:**
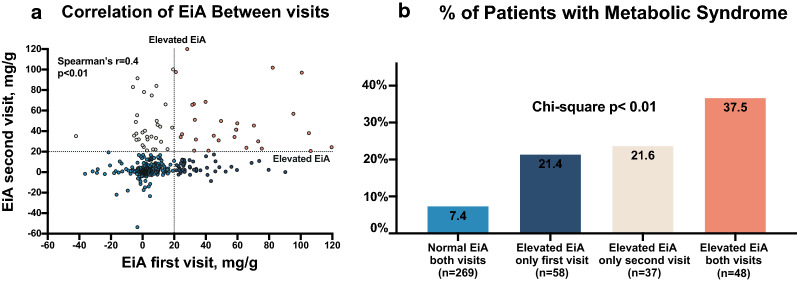
Exercise-induced albuminuria levels on two consecutive visits. **a** A scatter plot of exercise-induced albuminuria (EiA) levels during a participant’s first (X axis) and second (Y axis) visit. Dotted lines mark the threshold for elevated EiA (> 20 mg/g, bottom limit of top quartile). P value by spearman’s r test. Point color marks are according to the four groups shown in **b**. **b** Bar graph depicting the percent of patients diagnosed with metabolic syndrome during their first visit, according to their EiA status (elevated vs normal levels). Colors correspond to the scatter plot in **a**. p value by Chi square. *EiA* Exercise-induced albuminuria

EiA measurements demonstrated a significant correlation between the two visits (Fig. 1a). Elevated EiA (> 20 mg/g) at only one time point was associated with increased prevalence of metabolic syndrome diagnosis (Fig. 1b). Moreover, subjects with consistent elevated EiA on both visits were more likely to have a metabolic syndrome diagnosis during their first visit (Fig. 1b). These findings strengthen our past reports linking increased levels of exercise induced albumin excretion to metabolic dysfunction [7, 22]. Notably, patients that had elevated EiA only on their first visit and not during follow-up, demonstrated a greater reduction in fasting blood glucose between visits compared with the rest of the cohort; change in blood glucose = − 3 mg/dl [− 8.5 to 2.3] vs − 1 [− 7 to 5] for median [IQR] (p = 0.03) (Table 1).

### Diabetes/impaired fasting blood glucose and EiA levels over time

**Fig. 2:**
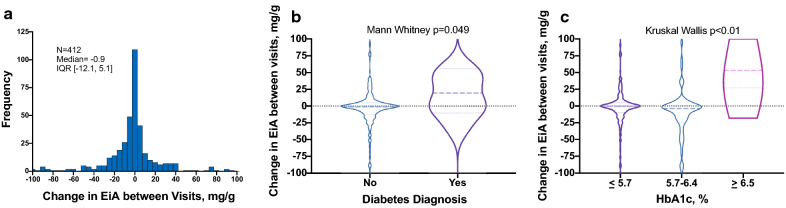
Change in exercise-induced albuminuria levels over time. **a** A histogram of the change in exercise-induced albuminuria (EiA) levels among the entire cohort (i.e. EiA on second visit minus EiA at baseline) (n = 412). **b** Violin plot of the change in EiA in patients with and without diagnosed diabetes during their first visit (n = 13 and 399). Diabetic patients increased their EiA over time. p by Mann–Whitney test. **c** Violin plot of the change in EiA according to categorized HbA1c levels; healthy (≤ 5.7% n = 331), pre-diabetic (5.7–6.4% n = 72) and diabetic individuals (≥ 6.5% n = 9). p by Kruskal–Wallis test. *EiA* Exercise-induced albuminuria, *HbA1c* Hemoglobin A1c

Next, we sought to characterize the change in EiA over time. The majority of the cohort demonstrated a minor change in EiA levels between the two visits; median change in EiA = − 0.9 [− 12.3 to 5.1] [IQR] (Fig. 2a). However, patients with a past diabetes diagnosis and/or subjects with HbA1c ≥ 6.5% significantly increased their EiA over time (Fig. 2b, c). Accordingly, diabetic patients had higher EiA levels on their second visit compared with non-diabetic controls; 31.9 [7.4–145.1] vs 2.4 [0–12.4] for median [IQR] (p < 0.01). The change in rest UACR between the two visits was not significantly different in patients with and without diabetes; − 0.7 [− 3.9 to 19.3] vs − 0.4 [− 4.5–1.7] for median [IQR] (p = 0.4).

A diabetes diagnosis was significantly associated with a high increase in EiA over time (top 10th percentile) even after adjusting for age, BMI, eGFR, METs, self-reported history of heart disease, systolic and diastolic blood pressure; OR = 4.4 (1.01–19.3 95% CI) (p = 0.049). Moreover, the continuous change in fasting blood glucose concentration between visits was the only significant predictor of a high increase in EiA over time after adjusting for age, sex and the respective changes in BMI, eGFR, systolic and diastolic blood pressure, and METs; OR = 1.05 (95% CI 1.01–1.09) (p = 0.03).

**Fig. 3:**
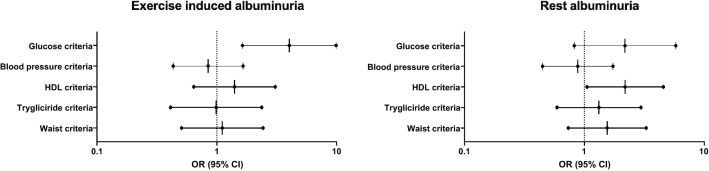
Multivariate analysis of metabolic syndrome components to predict a high increase in EiA and rest albuminuria over time. We ran a multivariate binary logistic regression to predict a high increase in EiA (left side) and rest albuminuria (right side) over time (top 10th percentile). The forest plots present the odds ratio (OR) and 95% confidence intervals of each metabolic syndrome component (see definition criteria in “Methods” section). Glucose criteria (elevated fasting blood glucose) was the only significant predictor in the EiA model (OR = 4.0 95% CI = 1.6–9.8, p < 0.01) while presence of the HDL-cholesterol criteria precited a greater increase in rest measurements (OR = 2.2 95% CI = 1.01–4.6, p = 0.04). *EiA* Exercise-induced albuminuria, *UACR* Urinary albumin to creatinine ratio

Finally, we wanted to determine which parameters of the metabolic syndrome were associated with the observed increase in EiA levels over time. Elevated fasting blood glucose criteria (≥ 100 mg/dl) was the strongest and only significant predictor for an elevated increase in EiA over time after adjusting for all five metabolic syndrome components; blood glucose, waist circumference, blood triglycerides, HDL cholesterol, and blood pressure criteria (Fig. 3). When comparing EiA to rest albuminuria measurements, elevated blood glucose was not significantly associated with increased changes in rest UACR, while decreased HDL cholesterol was the only significant predictor in the rest model (Fig. 3).

Notably, although patients with hypertension had higher EiA levels compared with normotensive subjects on both visits (median [IQR] = 8 mg/g [0–32] vs 3.5 mg/g [− 0.2 to 15.8] (p = 0.04) on the first visit and 4.2 [0.3–19.2] vs 2.2 [− 0.2 to 12.3] (p = 0.08) on the second visit), they did not change their EiA levels over time differently than normotensive individuals; change in EiA = − 1.9 [− 17.9 to 5.9] vs. − 0.8 [− 10.9 to 5.1] for median [IQR] (p = 0.36) respectively. Hypertensive patients taking antihypertensive medications (n = 58) had a greater reduction in EiA between the two visits compared with untreated individuals, albeit not statistically significant; change in EiA between visits = − 3.1 mg/g [− 24.1 to 11.5] vs. − 1.6 mg/g [− 12.9 to 4.2] for median [IQR] (p = 0.7). The majority of treated patients used angiotensin converting enzyme inhibitors (ACEi) and/or angiotensin receptor blockers (ARBs) (70%) (Table 1). ACEi and ARBs have been shown to reduce albuminuria levels [24] and this might explain the greater reduction in EiA over time among treated patients.

## Discussion

The main finding of this study is that patients with diabetes and/or elevated fasting blood glucose increase their exercise-induced urinary albumin excretion over time. Metabolic dysfunction was associated with consistent elevated levels of exercise induced albuminuria in a cohort of young and apparently healthy individuals without overt kidney disease.

Excessive urinary albumin excretion following exercise has been reported in patients with metabolic syndrome [7], including patients with hypertension [8] and type 2 diabetes [9, 10]. We present here, for the first time, a prospective study that characterizes EiA variation over time in patients with and without metabolic dysfunction. Our findings show that EiA levels are relatively stable among healthy individuals and do not change significantly over a mean follow-up period of 3 years. However, metabolic dysfunction, and more specifically impaired glucose metabolism seems to drive an increase in urinary albumin excretion following exercise which continues to elevate over time. These differences between patients with and without altered glucose metabolism were not seen using standard rest albuminuria measurements which stayed relatively stable during follow up.

The current study is in line with our past reports on urinary albumin excretion following exercise and its association with multiple risk factors for CVD [8, 22, 25]. Measuring EiA is a potential new sensitive and early biomarker for the kidney’s “reservoir capacities”, endothelial dysfunction and other metabolic related end organ damage. Results of future reports from this study cohort and others on the ability of EiA to predict major renal and cardiovascular events are highly anticipated. Whether EiA measurements provide additional clinical utility in stratifying risk as opposed to standard rest UACR calculations is still unknown.

A reduction in albuminuria is related to favorable cardiovascular and renal outcomes, especially in patients with diabetes [26–28]. While novel treatment strategies for cardio-renal outcomes such as SGLT-2 inhibitors are effective in lowering rest albuminuria [29, 30], their effect on exercise induced excretion of albumin is not entirely clear. We suggest that monitoring exercise induced levels of albuminuria may serve as a screening tool for pre-diabetic and diabetic individuals who require early initiation of such renal protective therapies [31, 32].

Our study has several limitations. First, the relatively low prevalence of pre-existing co-morbidities in our cohort limit our ability to describe the kinetics of EiA in patients at high risk for CVD or patients with overt kidney disease. Future prospective studies in older patients with evident CVD and renal disease are needed to better define the “normal” range of EiA and its variation over an individual’s life span. Furthermore, the majority of our study population improved their metabolic profile during follow up (reduced BMI, total cholesterol and triglycerides) which may imply that these individuals adopted a healthier life style after their first visit to our annual heath survey. This trend cannot necessarily be generalized to other populations. Nonetheless, the group of individuals who regressed their EiA from abnormal values at baseline to normal values on the second visit were the ones who had the greatest reduction in fasting blood glucose levels. This strengthens our main hypothesis that glucose metabolism is the main predictor of EiA in presumably healthy adults. A second limitation is that part of our observed changes in EiA over time could be attributed to the ‘regression to the mean phenomenon’ [33] which needs to be accounted for whenever assessing changes in a biological measurement over time. And yet, although patients with diabetes have consistent higher levels of EiA compared with normo-glycemic individuals [22], they still demonstrated a higher increase in EiA over time. The majority of diabetic patients did not “regress” down to the mean, thus highlighting the presence of a true biological driver for increasing EiA in this sub-population.

It should be noted that our current threshold for an increased change in EiA (top 10th percentile) was statistically driven in order to study the extremities of the cohort and will have to be adjusted in future prospective studies that will measure clinical outcomes.

Finally, the current study cohort was notably male dominant (85% men) and limited our ability to determine statistically significant sex-and-gender related differences in EiA levels over time. Our past report shows that elevated EiA is associated with abnormal ECG findings in apparently healthy women rather than men [22]. Thus, future studies should strive towards implementing sex-specific thresholds for EiA levels which may lead to better risk stratification of kidney and CVD.

## Conclusion

We conclude that individuals with diabetes and/or elevated fasting blood glucose increase their EiA levels over time. EiA should be further studied as an early and sensitive biomarker for kidney reserve and endothelial dysfunction in patients with metabolic syndrome.

## Data Availability

The data that support the findings of this study are available from the corresponding author upon reasonable request.

## Abbreviations

BMI: Body mass index
CVD: Cardiovascular disease
EiA: Exercise induced albuminuria
HbA1c: Glycated hemoglobin
ECG: Electrocardiogram
UACR: Urinary albumin to creatinine ratio
eGFR: Estimated glomerular filtration rate
HDL: High-density lipoprotein-cholesterol
LDL: Low-density lipoprotein-cholesterol
METs: Metabolic equivalents score
SD: Standard deviation
ANOVA: Analysis of variance
CI: Confidence interval
OR: Odds ratio
IQR: Inter quartile range
